# Water intake, hydration status and cognitive functions in older adults – a pilot study

**DOI:** 10.1007/s00394-025-03690-1

**Published:** 2025-05-09

**Authors:** Agata Białecka-Dębek, Dawid Madej, Emilia Łojek

**Affiliations:** 1 Department of Human Nutrition, Institute of Human Nutrition Sciences, Warsaw University of Life Sciences - SGGW, Warsaw, Poland; 2 Faculty of Psychology, University of Warsaw, Warsaw, Poland

**Keywords:** Plasma osmolality, Urine osmolality, Extracellular water, Cognitive domains, Dehydration

## Abstract

**Purpose:**

This study aimed to assess the relationship between the hydration status and cognitive functioning of older adults. The novelty of the study was the simultaneous use of several indicators of hydration status, including plasma and urine osmolality, specific gravity and urine color, as well as the assessment of total body water content from body composition measurements, together with comprehensive cognitive assessment.

**Methods:**

A cross-sectional pilot study included 35 participants aged ≥ 60 years. Water intake was assessed using the 3-day food record method. Hydration status was assessed by plasma osmolality (Posm), urine osmolality (Uosm), specific gravity (USG) and color (UC), extracellular water (ECW) and percentage of total body water (%TBW). Cognitive functions were assessed using a set of standardized neuropsychological tests including: two verbal tests (Digit Span, DS and Vocabulary, VT) from the Wechsler Adult Intelligence Scale, California Verbal Learning Test (CVLT), Verbal Fluency Test (VFT), Grooved Pegboard Test (GPT) and Global Cognitive Function (GCF).

**Results:**

The %TBW was the most strongly related to cognitive processes of all the measures of hydration status. %TBW was significantly related to the performance on memory/learning based on CVLT (*r* = -0.55, *p* = 0.002), after a short delay (*r* = -0.59, *p* = 0.001) and long delay (*r* = -0.57, *p* = 0.001) and GCF (*r* = -0.43, *p* = 0.019). Marked correlations were also present between %TBW and psychomotor speed using the GPT (*r* = 0.41, *p* = 0.028). Moreover, significant relationships were obtained in cluster analyses. Cluster 2 (lower hydration status) was characterized by lower water intake and AI% (% of Adequate Intake), higher Uosm, USG, UC, ECW and %TBC than cluster 1. At the same time, it had significantly higher scores for language ability: VT (*p* = 0.041) and VFT (*p* = 0.041).

**Conclusion:**

Significant relationships between some indicators of hydration status and selected cognitive domains were observed. This pilot study complements previous research on the relationship between hydration status and cognitive function in older adults, emphasizing that even small changes in hydration status assessment parameters can affect cognitive outcomes. In healthy, free-living older adults without dehydration assessed by plasma osmolality, other parameters of hydration status, such as water intake and urine parameters, influence language functions, suggesting the need to assess multiple markers simultaneously. The long-term effect of low water intake should be evaluated in a larger study group.

## Introduction

Aging is an inevitable and progressive biological process that leads to irreversible physiological and functional changes throughout the body [1]. With age, the functions of many organs and systems deteriorate, particularly the cardiovascular, respiratory, digestive, and renal systems [1, 2]. The aging process also leads to changes in the nervous system, with consequent changes in cognitive functioning, including: slower information processing, problems with complex attention, learning new information, visuospatial/construction, and executive functions. Overall, language ability and general knowledge remain intact with aging [3]. Achieving old age in good health without symptoms of neurocognitive decline results from a complex interaction of many different factors [4, 5]. The literature emphasizes the importance of nutritional factors, ranging from daily diet supplementation with specific nutrients and changes in dietary habits to attention to overall dietary patterns. It also highlights the gap in investigating other aspects of nutrition (including hydration status and the chronobiology of food intake) that may explain the hidden links between nutrition and neurocognitive dysfunction [6].

Older adults are at risk of dehydration, with studies suggesting a prevalence of 20–30% in this population [7]. Dehydration refers to the process of losing water from the body [8]. This can be caused by not drinking enough (low-intake dehydration), by excessive loss (through bleeding, vomiting, diarrhea, etc., called volume depletion), or by a combination of the two (combined dehydration). Low-intake dehydration is a shortage of pure water that leads to both intracellular and extracellular fluid loss and increased osmolality in both compartments (intracellular and extracellular) [9]. Dehydration is associated with increased mortality, morbidity, and disability in older people [7]. Inadequate water intake leads to deterioration in neurocognitive and other neurological functions. In a study of mice deprived of access to water for 24–48 h, an increase in plasma osmolality and vasopressin levels was observed, as well as an increase in the production of reactive oxygen species (ROS). In addition, dehydration reduced nerve-activated blood flow and response to vasodilators. Dehydration-induced cerebral vascular dysfunction has been associated with cognitive deficits using the Y maze [10]. The results of studies assessing the relationship between hypohydration and neurocognitive functions in humans provide inconsistent results and differ in methodological approach [11]. The meta-analysis results showed that even in healthy young adults, a loss of more than 2% of body mass may impair attention, executive functions, and motor coordination, but not reaction time [12]. On the other hand, another meta-analysis study shows that mild dehydration does not significantly affect cognitive performance in healthy young adults but may markedly affect mood [11]. Older populations have been suggested to be particularly vulnerable to dehydration, with greater detrimental effects [13]. However, there has been very little investigation into the effects of mild dehydration on neurocognitive functioning [13]. One of the major challenges is the lack of a sensitive marker of hydration status in older adults. Various single methods to assess hydration status have been used in different studies, and the methodological heterogeneity makes the results difficult to compare [14].

Thus, this study aimed to investigate the relationship between hydration status and cognitive functions in older adults. Previous studies have used single methods to assess hydration status, and methodological heterogeneity makes it difficult to compare results. To the best of our knowledge, this is the first study to compare different methods of assessing hydration status in relation to cognitive functions in older adults. Among the markers used in the study were plasma and urine osmolality, specific gravity and urine color, and assessment of total body water content from body composition measurements.

## Materials and methods

### Participants and study design

A cross-sectional pilot study of 35 free-living (noninstitutionalized) participants (66% women) aged 61–77 (mean 68.7 ± 4.1 years) was conducted. Information about opportunities to participate in the study was distributed by leaflets and was published in the local paper. Participants took part in the study voluntarily. Each participant was informed about the purpose of the study, the methodology used, and the right to withdraw from participation without any consequences. Voluntary participation was documented by signing an informed consent form to participate in the study. The study’s inclusion criteria required individuals to be over 60, exhibit independence, and provide informed consent. Exclusion criteria included: the diagnosed presence of advanced neoplastic disease, neurodegenerative disease (including dementia), depression; renal failure or damage; chronic use of diuretics and laxatives; history of severe head injuries; malnutrition (Body Mass Index - BMI less than 18.5 kg/m^2^); fever, diarrhea, or vomiting in the week before testing. The study protocol was approved by the Human Research Ethics Committee of the Faculty of Human Nutrition and Consumer Sciences at the Warsaw University of Life Sciences on 5 July 2018, Resolution No. 02p/201. The research was conducted in Poland (Central Europe) during the autumn/winter season.

Diet and water intake were assessed using the 3-day food record method. Participants were given verbal and written instructions on how to complete the questionnaire. They were asked to note down all products, foods, and beverages consumed (including snacks and beverages consumed between meals, e.g. fruit, sweets, salty snacks, coffee, beer, etc.) with the following information referring to them: the commercial name of the product (e.g. Baltona bread, cottage cheese, etc.), the quality of the product (e.g. wheat bread, 1.5% fat milk, Gouda cheese), the origin of the dish (e.g.: commercial dish) or the exact recipe of the dish, including the types of additives used in the dish (also for meals) and note any added sugar, salt or fat and the method of preparation (e.g.: boiled in water, steamed, baked, fried, stewed with/without fat, grilled, etc.). The respondents were also asked to state the portion size of each product, food, and beverage consumed as accurately as possible, using a kitchen scale and expressing in-home or commercial measures for verification. The records were then complemented with a dietitian interview. All participants were given a digital kitchen scale (Clatronic KW 3412, 1 g accuracy). Daily water intake was calculated using the Dieta 6. software and compared with reference values for adequate intake (AI = 2000 ml for women and 2500 ml for men) [15].

Non-nutritional factors affecting cognitive functions, e.g., age, education level, health status, physical, mental, and social activity, and selected lifestyle parameters (smoking, alcohol consumption, dietary supplements) were assessed using a questionnaire method. The level of physical activity was categorized as low (including a sedentary lifestyle, sometimes short walks or other low activity and low-intensity exercise i.e. exercise that does not involve sweating and that can be done while talking to another person, such as walking, dancing, fishing or hunting, shopping without using a car, at least 2–4 h per week), moderate (moderate-intensity exercise, i.e. exercise that involves sweating and that cannot be done while talking to another person, such as running, walking uphill, swimming, gymnastics, digging in the garden or field, cycling uphill, etc., or low-intensity exercise for more than 4 h per week), and high (intense exercise with maximum tolerable effort regularly several times per week). In addition, blood pressure measurement was taken using the standard procedure in a sitting position with an automatic blood pressure monitor (Omron M2 Basic).

### Cognitive functions

The Polish adaptations of standardized neuropsychological tests, known as valuable measures for assessing cognitive functions in older adults, were employed [16]. The battery consisted of the following methods:

The Mini-Mental State Examination (MMSE) is a clinical screening scale for assessing cognitive impairment. It is a 30-point test that quantitatively assesses various aspects of cognitive functioning [17, 18]. The MMSE was administered at the time of recruitment, and participants who scored less than 24 points (indicating dementia) were not included in the study.

The California Verbal Learning Test (CVLT) measures the ability to learn and remember verbal material. The test consists of two 16-word lists: List A and a distraction List B. The subject has to learn the List A by repeating it five times immediately after presentation, then is asked to recall it after repeating the List B (a short delay; CVLT-s), and after a 20-minute break (a long delay; CVLT-l). The total score (CVLT-t) includes the number of words correctly recalled in five trials of List A. The maximum possible score was 80; a higher score indicates better cognitive functioning. The Polish standardization of the CVLT demonstrated stability, and correlations with measures of cognitive function confirmed validity in the between-group comparisons [19, 20].

The Color Trails Test (CTT) assesses attention and executive functions, in particular perceptual tracking, sustained and divided attention, sequencing and self-monitoring. Psychomotor skills are also involved. The CTT consists of two parts, CTT-1 and CTT-2, administered consecutively. Each part contains pink and yellow circles with numbers from 1 to 25, and the subject connects the circles with numbers from 1 to 25 in sequence with straight lines, alternating between the pink and yellow colors in the CTT-2. The scores are the completion times (in seconds) for each part. Due to the need to switch from one color sequence to another, executive functions are involved in the execution of the CTT-2 to a much greater extent than in the CTT-1. The Polish standardization confirmed the stability of CTT indicators, and the consistency of clinical interpretations as high [21, 22].

The Grooved Pegboard Test (GPT) is a manipulative dexterity test measuring psychomotor speed and complex visual-motor coordination [16]. In this test, metal pegs should be inserted into 25 holes with randomly arranged slots on a pegboard as quickly as possible. The task is performed three times with the dominant hand (GPT-d) and three times with the non-dominant hand (GPT-n). The scores (the time in seconds) for each hand are averaged.

The Digit Span Test (DST) from the WAIS-R Adult Intelligence Scale measures working memory and has been comprised: DST Forward (DST-f) and DST Backward (DST-b). DST-f requires the repetition of numbers in the same order as read aloud by the examiner, and DST-b requires the repetition of numbers in the reverse order as presented by the examiner [23].

The Vocabulary Test (VT) from the WAIS-R Adult Intelligence Scale measures the knowledge of word meanings. VT consists of 35 produce-the-definition items. Participants are given a target word and asked to define it. Complete definitions receive an item score of 2, while incomplete definitions receive a partial credit item score of 1. The total score is the sum of the item scores. Results were given as raw data, with a higher score indicating better cognitive functioning [23].

The Verbal Fluency Test (VFT), measuring verbal skills and cognitive flexibility, tests executive functions [16]. The task is to say as many words as possible from a specific category (semantic fluency) or words starting with a specific letter (phonetic fluency) in one minute. The Polish version of the VFT used in this study [24] consisted of five word naming trials: generating words from the Animals, Fruits and Verbs categories and words starting with letters K and F. The sum of correctly named words in all trials was analysed.

All of the above tests except VT measure cognitive functions sensitive to aging and are referred to as indices of fluid intelligence [16]. VT is considered one of the best indicators of crystallized intelligence, relatively resistant to aging [3, 16].

To assess overall cognitive function, a Global Cognitive Function (GCF) score was determined as the outcome measure. Raw scores for each individual cognitive assessment were standardized using the mean and standard deviation as normative data, creating z-scores (Z). GCF was calculated as a composite z-score of 8 tests, adding or subtracting each individual test value based on whether a higher score indicates higher or lower cognitive performance, respectively, as the formula:

$$\begin{gathered}GCF = ({Z_{CVLT - t}}\: + ( - {Z_{CTT1}}\:) \hfill \\+ ( - {Z_{CTT2}}\:) + ( - {Z_{GPT - d}}\:) \hfill \\+ ( - {Z_{GPT - n}}\:) + {Z_{DST}}\: \hfill \\+ \:{Z_{VT}}\: + \:{Z_{VFT}})\:/\:8 \hfill \\\end{gathered} $$

### Hydration status

Hydration status was assessed using urine, blood, and body composition assessments. The study was designed in such a way that there was no additional variable factor in the form of seasonal changes. Data were collected only during the autumn-winter season.

Urine samples were collected from participants using the first morning urine, following detailed instructions provided to them. The samples were obtained on the day of cognitive testing, then immediately frozen and stored at -80 °C for further analysis. The maximum storage time was six months. Urine markers included: urine specific gravity (USG), urine osmolality (Uosm), and urine color (UC). USG was measured by the refractometric method (Digital Hand-held Compact Refractometer PAL-10 S ATAGO) and performed at optimal temperature (20–22 °C). Uosm was measured by freezing point osmometry (Osmometer Marcel OS 3000). UC assessment was based on the original urine eight-color sampler. All samples were tested in the same environment, in identical containers, with the same volume of urine, and by one operator.

Blood was taken from an ulnar vein by a qualified nurse at a medical clinic, where medical care was also provided. Participants attended the laboratory after fasting overnight. Blood samples were collected and analyzed in a certified laboratory the day after cognitive testing. Plasma osmolality (Posm) was measured by the Osmometr OS 300. A comprehensive blood panel was conducted to assess various aspects of health. This included a general blood count to evaluate overall health and blood cell status. Metabolic health and nutrition were assessed by measuring glucose, sodium, potassium, 25-hydroxyvitamin D, a lipid profile (including cholesterol and triglycerides), folates, vitamin B12, and homocysteine. Markers of inflammation and immune response, such as high-sensitivity C-reactive protein (hsCRP) and interleukin 6 (IL-6), were also measured. Finally, kidney function was evaluated using glomerular filtration rate (GFR) protein levels, creatinine, and urea.

During the day of cognitive testing, body composition analysis, including measurement of total body water (TBW), intracellular water (ICW), and extracellular water (ECW), was performed using the bioelectrical impedance method (resistance at 50 kHz, InBody 770 Composition Analyzer). Body weight and height were also measured to calculate Body Mass Index (BMI). The variables used in the study were: (1) percent of total body water by weight (%TBW = TBW/WT*100%), and (2) ECW/TBW ratio.

### Statistical analysis

The statistical analyses were performed using Statistica software (version 13.4, StatSoft, US). The characteristics data were presented as percentages of participants. The Chi-square test was used to determine statistically significant differences between categorical variables and sex groups. Age, BMI, energy intake, selected nutrients, and hydration status were shown as mean values ± standard deviation (SD). The hypothesis of normality of continuous variables was rejected based on the Shapiro-Wilks test; therefore, the Mann-Whitney U tests were used to determine statistically significant differences between sex groups (p-values of ≤ 0.05 were admitted as statistically significant). Pearson correlation coefficients (after transformation of the data (log(x)) to obtain normal distributions) were used to determine associations between hydration status and cognitive function tests. Partial correlations were assessed to take into account for the potential effect of the following parameters on the observed associations: age (years, continuous), BMI (kg/m^2^, continuous), education (< 16 years, ≥ 16 years), smoking (ever, never), alcohol consumption (ever, never), and energy (kcal/d, continuous). Cluster analysis was performed using the k-means method (a posteriori analysis). The observation was selected to maximize cluster distances. The input variables for the analysis included: water intake, Posm, USG, and ECW/TBC. All input variables were standardized to achieve a mean equal to 0 and a standard deviation equal to 1 and expressed as Z-score values. A division into two clusters was adopted. The identified clusters comprised 48.6% and 51.4% of the participants, respectively. The Mann-Whitney U tests assessed differences in mean cognitive test scores between the two clusters.

## Results

### Characteristics of the study population

The characteristics of the studied population by sex are presented in Table 1. The average age of the study population was 68.7 ± 4.1 years; there was no statistically significant difference between men and women. Men consumed significantly more sodium and protein than women (*p* = 0.005 and *p* = 0.023, respectively). The intake of the other components did not differ significantly between the groups. The mean daily water intake (Table 1) from all sources (beverages and food products) in the study group was 2739 ± 529 ml/day. Adequate water intake (above AI reference values) was found in 83% of the subjects. The mean Uosm in the study group was 477.26 ± 193.86 mOsm/kg, with a higher value indicating poorer hydration status. The mean value of USG in the study group was 1.014 ± 0.006, with a value below 1.010 indicating excessive fluid intake (26% of people in the study group) and above 1.020 indicating dehydration [25] (20% of people in the study group). The mean % TBW was 48.3% in the total study group, 52.9% in the male, and 45.7% in the female (*p* = 0.001). Moreover, the mean Posm in the study group was 293 ± 5 mOsm/kg. A value above 300 mOsm/kg indicates current low-intake dehydration [26].

**Table 1:** Demographic, lifestyle, dietary, and hydration characteristics of study sample (mean ± SD)

Variables	Total (*n* = 35)	Women (*n* = 23)	Men (*n* = 12)	*P*-value
Age *(years)*	68.7 ± 4.1	68.4 ± 4.3	69.4 ± 3.8	0.555 ^a^
BMI *(kg/m*^*2*^*)*	27.4 ± 4.2	27.3 ± 4.5	27.5 ± 3.5	0.664 ^a^
Physical activity *(%)*				
* Low*	51.4	47.8	58.3	0.679 ^b^
* Moderate*	42.9	47.8	33.3	
* High*	5.7	4.3	8.3	
Regular smoking *(%)*	42.9	52.2	25.0	0.123 ^b^
Alcohol consumption *(%)*	85.7	82.6	91.7	0.467 ^b^
Supplement use *(%)*	82.9	91.3	66.7	0.066 ^b^
Drugs use *(%)*	74.3	73.9	75.0	0.944 ^b^
Chronic diseases (%)	68.6	65.2	75.0	0.554 ^b^
Education time *(%)*				
* < 16 years*	34.3	34.8	33.3	0.932 ^b^
* ≥ 16 years*	65.7	65.2	66.7	
Residence *(%)*				
* Urban area*	97.1	95.7	100	0. 464 ^b^
* Suburban area*	2.9	4.3	0	
Lives alone *(%)*	40	57	8	0.012 ^b^*
Social contacts *(%)*	100	100	100	1.0 ^b^
Energy intake *(kcal/d)*	1713 ± 434	1617 ± 345	1896 ± 537	0.170 ^a^
Sodium intake *(mg/d)*	3344 ± 1133	2950 ± 985	4098 ± 1040	0.005 ^a^*
Potassium intake *(mg/d)*	3181 ± 825	3078 ± 779	3379 ± 909	0.395 ^a^
Magnesium intake *(mg/d)*	360 ± 113	330 ± 76.9	416 ± 149	0.149 ^a^
Calcium intake *(mg/d)*	661 ± 223	615 ± 211	749 ± 227	0.092 ^a^
Protein intake *(g/d)*	77.3 ± 23.5	71.2 ± 22.1	89.0 ± 22.6	0.023 ^a^*
Fat intake *(g/d)*	68.0 ± 19.7	66.6 ± 18.6	70.8 ± 22.2	0.689 ^a^
Carbohydrate *(g/d)*	205 ± 69.1	190 ± 44.8	235 ± 96.4	0.149 ^a^
Water intake *(g/d)*	2739 ± 529	2811 ± 532	2602 ± 516	0.289 ^a^
%AI	128.0 ± 30.1	140.5 ± 26.6	104.1 ± 20.6	< 0.001 ^a^*
Uosm *(mOsm/kg)*	477 ± 194	386 ± 145	652 ± 154	< 0.001 ^a^*
USG	1.01 ± 0.01	1.01 ± 0.01	1.02 ± 0.01	< 0.001 ^a^*
UC	4.57 ± 1.59	3.78 ± 1.19	6.08 ± 1.08	< 0.001 ^a^*
%TBW	48.2 ± 6.5	45.7 ± 5.9	52.9 ± 4.7	0.001 ^a^*
ECW/TBW	0.39 ± 0.01	0.39 ± 0.01	0.39 ± 0.01	0.590 ^a^
Posm *(mOsm/kg)*	293 ± 5.2	293 ± 4.8	294 ± 6.1	1.000 ^a^

*p-value ≤ 0.05 (^a^ - Mann–Whitney *U* test, ^b^ - Chi^2^ test)

AI, Adequate Intake; BMI, body mass index; ECW, extracellular water; Posm plasma osmolality; TBW, total body water; UC, urine colour; Uosm, urine osmolality; USG, urine specific gravity

### Correlation coefficients between parameters of hydration status

Total water intake did not correlate with markers of hydration status or with the results of cognitive test scores. However, the %AI ​​level was negatively correlated with all urine parameters, Uosm (*r* = -0.60, *p* = 0.001), USG (*r* = -0.59, *p* = 0.001), and UC (*r* = -0.49, *p* = 0.007). The analyses showed a significant correlation between Posm and serum urea levels (*r* = 0.42, *p* = 0.028), but no significant relationship was found between this marker and other hydration status markers. Uosm correlated positively with USG (*r* = 0.95, *p* < 0.001) and UC (*r* = 0.73, *p* < 0.001) (data not shown in tables).

### Correlation coefficients between hydration status and cognitive functions tests

Correlations between markers of hydration status and results of cognitive assessment tests are shown in Table 2. Total water intake and Posm did not correlate with the results of the cognitive test scores in the total study group (Table 2) or by sex (Tables 3 and 4). Uosm, USG, and UC did not correlate with cognitive test scores in the total group. However, in women, Uosm was positively correlated with CVLT-t (*r* = 0.59, *p* = 0.014), and USG was positively correlated with CVLT-t (*r* = 0.61, *p* = 0.010), CVLT-s (*r* = 0.49, *p* = 0.043) and CVLT-l score (*r* = 0.49, *p* = 0.047). UC was positively correlated with CVLT-t score in women (*r* = 0.59, *p* = 0.013) and in men (*r* = 0.85, *p* = 0.034) but negatively correlated with DST score in men (*r*=-0.93, *p* = 0.007). Mean %TBW was negatively correlated with CVLT-t (*r* = -0.55, *p* = 0.002), CVLT-s (*r* = -0.59, *p* = 0.001), CVLT-l (*r* = -0.57, *p* = 0.001) and GCF (*r* = -0.43, *p* = 0.019) in the total population and this relationship was visible in women (respectively, *r* = -0.57, *p* = 0.017; *r* = -0.64, *p* = 0.005; *r* = -0.53, *p* = 0.028; *r* = -0.50, *p* = 0.042). Additionally, in women, %TBW was negatively correlated with VFT (*r* = -0.56, *p* = 0.019). Positive correlations were observed between %TBW and GPT-d in the total group (*r* = 0.41, *p* = 0.028) and males (*r* = 0.95, *p* = 0.004). ECW/TBW ratio was negatively correlated with GCF in the total population (*r* = -0.38, *p* = 0.042) and in women (*r* = -0.50, *p* = 0.042).

**Table 2:** Pearson correlation coefficients between hydration status and cognitive function tests (r, *p*)

Variables	Water intake		Uosm		USG		UC		%TBW		ECW/TBW		Posm	
	Crude	Partial^a^	Crude	Partial^a^	Crude	Partial^a^	Crude	Partial^a^	Crude	Partial^a^	Crude	Partial^a^	Crude	Partial^a^
CVLT-t	0.03 *p* = 0.875	0.07 *p* = 0.738	-0.15 *p* = 0.392	-0.08 *p* = 0.667	-0.07 *p* = 0.673	-0.05 *p* = 0.792	-0.05 *p* = 0.772	-0.04 *p* = 0.846	-0.14 *p* = 0.411	-0.55 *p* = 0.002*	0.04 *p* = 0.842	0.19 *p* = 0.329	-0.05 *p* = 0.755	-0.19 *p* = 0.311
CVLT-s	-0.02 *p* = 0.906	0.03 *p* = 0.870	-0.05 *p* = 0.781	-0.05 *p* = 0.777	-0.03 *p* = 0.871	-0.06 *p* = 0.772	-0.06 *p* = 0.746	-0.10 *p* = 0.589	-0.11 *p* = 0.517	-0.59 *p* = 0.001*	-0.05 *p* = 0.774	0.06 *p* = 0.773	-0.28 *p* = 0.107	0.07 *p* = 0.707
CVLT-l	0.07 *p* = 0.689	0.07 *p* = 0.735	-0.08 *p* = 0.642	-0.04 *p* = 0.832	-0.03 *p* = 0.855	-0.03 *p* = 0.865	-0.02 *p* = 0.897	-0.10 *p* = 0.600	0.01 *p* = 0.975	-0.57 *p* = 0.001*	0.02 *p* = 0.888	0.22 *p* = 0.251	-0.13 *p* = 0.470	-0.10 *p* = 0.617
CTT1	-0.06 *p* = 0.733	-0.06 *p* = 0.767	-0.05 *p* = 0.789	0.06 *p* = 0.751	-0.12 *p* = 0.510	-0.03 *p* = 0.863	-0.07 *p* = 0.706	-0.03 *p* = 0.882	0.08 *p* = 0.634	0.03 *p* = 0.861	0.17 *p* = 0.320	0.15 *p* = 0.438	-0.32 *p* = 0.059	0.35 *p* = 0.062
CTT2	0.02 *p* = 0.925	-0.01 *p* = 0.943	-0.08 *p* = 0.647	0.08 *p* = 0.697	-0.09 *p* = 0.607	0.02 *p* = 0.899	-0.12 *p* = 0.502	-0.08 *p* = 0.675	0.30 *p* = 0.083	0.09 *p* = 0.638	0.36 *p* = 0.035*	0.26 *p* = 0.180	-0.01 *p* = 0.972	0.07 *p* = 0.722
GPT-d	-0.07 *p* = 0.709	-0.01 *p* = 0.954	0.27 *p* = 0.119	0.35 *p* = 0.060	0.29 *p* = 0.091	0.36 *p* = 0.053	0.15 *p* = 0.397	0.23 *p* = 0.228	0.31 *p* = 0.071	0.41 *p* = 0.028*	0.51 *p* = 0.002*	0.44 *p* = 0.017*	0.06 *p* = 0.739	-0.06 *p* = 0.754
GPT-n	-0.12 *p* = 0.480	-0.06 *p* = 0.765	0.23 *p* = 0.189	0.31 *p* = 0.103	0.28 *p* = 0.106	0.36 *p* = 0.057	0.13 *p* = 0.448	0.23 *p* = 0.232	0.18 *p* = 0.294	0.34 *p* = 0.070	0.52 *p* = 0.001*	0.47 *p* = 0.011*	0.16 *p* = 0.355	-0.10 *p* = 0.589
DST	-0.15 *p* = 0.385	-0.23 *p* = 0.222	-0.09 *p* = 0.603	-0.01 *p* = 0.962	-0.02 *p* = 0.910	0.02 *p* = 0.922	-0.03 *p* = 0.874	-0.04 *p* = 0.835	0.02 *p* = 0.908	-0.05 *p* = 0.777	-0.22 *p* = 0.197	-0.12 *p* = 0.540	-0.25 *p* = 0.144	0.26 *p* = 0.170
VT	-0.22 *p* = 0.209	-0.18 *p* = 0.355	0.03 *p* = 0.884	0.07 *p* = 0.700	0.08 *p* = 0.642	0.09 *p* = 0.633	0.05 *p* = 0.780	-0.01 *p* = 0.952	0.16 *p* = 0.363	-0.20 *p* = 0.299	-0.23 *p* = 0.181	-0.15 *p* = 0.429	-0.24 *p* = 0.169	-0.04 *p* = 0.824
VFT	-0.13 *p* = 0.442	-0.15 *p* = 0.438	0.03 *p* = 0.876	0.07 *p* = 0.707	0.09 *p* = 0.622	0.09 *p* = 0.654	0.22 *p* = 0.206	0.17 *p* = 0.367	0.20 *p* = 0.261	-0.25 *p* = 0.191	-0.40 *p* = 0.017*	-0.29 *p* = 0.124	-0.42 *p* = 0.013*	0.10 *p* = 0.611
GCF	-0.05 *p* = 0.760	-0.07 *p* = 0.731	-0.12 *p* = 0.486	-0.18 *p* = 0.349	-0.06 *p* = 0.718	-0.14 *p* = 0.470	0.02 *p* = 0.908	-0.07 *p* = 0.726	-0.14 *p* = 0.421	-0.43 *p* = 0.019*	-0.52 *p* = 0.001*	-0.38 *p* = 0.042*	-0.19 *p* = 0.283	-0.10 *p* = 0.614

*p-value ≤ 0.05 ^a^In contrast to the crude (not adjusted) the partial correlation coefficients were adjusted for age (years, continuous), BMI (kg/m2, continuous), education (< 16 years, ≥ 16 years), smoking (ever, never), alcohol consumption (ever, never), and energy (kcal/d, continuous); AI, Adequate Intake; BMI, body mass index; CTT-1, Color Trails Test part 1; CTT-2, Color Trails Test part 2; CVLT-l, California Verbal Learning Test – long recall; CVLT-s, California Verbal Learning Test – short recall; CVLT-t, California Verbal Learning Test – sum; DST, Digit Span Test; ECW, extracellular water; GCF, Global Cognitive Function; GPT-d, Grooved Pegboard Test dominant hand; GPT-n, Grooved Pegboard Test non-dominant hand; Posm, plasma osmolality; TBW, total body water; UC, urine colour; Uosm, urine osmolality; USG, urine specific gravity; VFT, Verbal Fluency Tests; VT, Vocabulary Test

**Table 3:** Pearson correlation coefficients between hydration status and tests of cognitive function tests in women (r, *p*)

Variables	Water intake		Uosm		USG		UC		%TBW		ECW/TBW		Posm	
	Crude	Partial^a^	Crude	Partial^a^	Crude	Partial^a^	Crude	Partial^a^	Crude	Partial^a^	Crude	Partial^a^	Crude	Partial^a^
CVLT-t	-0.02 *p* = 0.922	-0.12 *p* = 0.649	0.42 *p* = 0.049*	0.59 *p* = 0.014*	0.45 *p* = 0.029*	0.61 *p* = 0.010*	0.41 *p* = 0.055	0.59 *p* = 0.013*	-0.06 *p* = 0.773	-0.57 *p* = 0.017*	-0.09 *p* = 0.678	0.03 *p* = 0.909	-0.08 *p* = 0.700	-0.38 *p* = 0.127
CVLT-s	-0.09 *p* = 0.681	-0.15 *p* = 0.567	0.37 *p* = 0.083	0.44 *p* = 0.077	0.43 *p* = 0.039*	0.49 *p* = 0.043*	0.27 *p* = 0.217	0.36 *p* = 0.159	-0.08 *p* = 0.702	-0.64 *p* = 0.005*	-0.07 *p* = 0.741	-0.02 *p* = 0.926	0.16 *p* = 0.454	0.02 *p* = 0.936
CVLT-l	0.02 *p* = 0.916	-0.10 *p* = 0.709	0.32 *p* = 0.142	0.44 *p* = 0.074	0.39 *p* = 0.069	0.49 *p* = 0.047*	0.29 *p* = 0.172	0.33 *p* = 0.200	0.06 *p* = 0.792	-0.53 *p* = 0.028*	-0.03 *p* = 0.883	0.15 *p* = 0.563	0.001 *p* = 0.985	-0.30 *p* = 0.235
CTT1	-0.23 *p* = 0.299	-0.22 *p* = 0.387	-0.03 *p* = 0.887	0.13 *p* = 0.612	-0.01 *p* = 0.965	0.13 *p* = 0.623	-0.02 *p* = 0.913	-0.13 *p* = 0.623	0.29 *p* = 0.183	-0.15 *p* = 0.572	0.13 *p* = 0.557	0.27 *p* = 0.304	0.34 *p* = 0.113	0.24 *p* = 0.364
CTT2	-0.14 *p* = 0.532	-0.23 *p* = 0.376	-0.11 *p* = 0.614	0.16 *p* = 0.536	-0.10 *p* = 0.652	0.14 *p* = 0.579	-0.11 *p* = 0.612	-0.01 *p* = 0.962	0.53 *p* = 0.009*	0.32 *p* = 0.214	0.38 *p* = 0.071	0.37 *p* = 0.141	-0.08 *p* = 0.719	-0.10 *p* = 0.716
GPT-d	-0.07 *p* = 0.759	-0.23 *p* = 0.381	-0.14 *p* = 0.532	0.03 *p* = 0.908	-0.17 *p* = 0.427	-0.01 *p* = 0.972	-0.46 *p* = 0.026*	-0.25 *p* = 0.338	0.31 *p* = 0.145	0.31 *p* = 0.226	0.74 *p* < 0.001*	0.75 *p* = 0.001*	-0.20 *p* = 0.363	0.03 *p* = 0.917
GPT-n	-0.19 *p* = 0.377	-0.18 *p* = 0.483	0.11 *p* = 0.631	0.19 *p* = 0.469	0.08 *p* = 0.732	0.17 *p* = 0.518	-0.16 *p* = 0.454	-0.06 *p* = 0.816	0.15 *p* = 0.505	0.41 *p* = 0.099	0.55 *p* = 0.006*	0.48 *p* = 0.051	-0.25 *p* = 0.243	-0.15 *p* = 0.572
DST	-0.10 *p* = 0.635	-0.09 *p* = 0.719	-0.18 *p* = 0.413	-0.23 *p* = 0.383	-0.16 *p* = 0.459	-0.21 *p* = 0.413	0.04 *p* = 0.869	-0.13 *p* = 0.610	-0.02 *p* = 0.920	-0.20 *p* = 0.448	-0.24 *p* = 0.277	-0.11 *p* = 0.682	0.27 *p* = 0.218	0.20 *p* = 0.443
VT	-0.13 *p* = 0.552	-0.11 *p* = 0.662	0.25 *p* = 0.251	0.35 *p* = 0.163	0.29 *p* = 0.173	0.40 *p* = 0.114	0.18 *p* = 0.406	0.11 *p* = 0.663	0.15 *p* = 0.505	-0.31 *p* = 0.226	-0.26 *p* = 0.227	-0.24 *p* = 0.357	-0.12 *p* = 0.571	-0.12 *p* = 0.634
VFT	-0.12 *p* = 0.582	-0.10 *p* = 0.703	0.21 *p* = 0.339	0.26 *p* = 0.322	0.25 *p* = 0.245	0.28 *p* = 0.280	0.26 *p* = 0.226	0.14 *p* = 0.579	0.12 *p* = 0.585	-0.56 *p* = 0.019*	-0.49 *p* = 0.018*	-0.42 *p* = 0.094	0.16 *p* = 0.471	-0.04 *p* = 0.878
GCF	0.04 *p* = 0.841	0.08 *p* = 0.765	0.19 *p* = 0.399	0.09 *p* = 0.735	0.22 *p* = 0.314	0.12 *p* = 0.637	0.32 *p* = 0.136	0.22 *p* = 0.408	-0.22 *p* = 0.306	-0.50 *p* = 0.042*	-0.58 *p* = 0.003*	-0.50 *p* = 0.042*	-0.27 *p* = 0.217	-0.21 *p* = 0.424

*p-value ≤ 0.05 ^a^In contrast to the crude (not adjusted) the partial correlation coefficients were adjusted for age (years, continuous), BMI (kg/m2, continuous), education (< 16 years, ≥ 16 years), smoking (ever, never), alcohol consumption (ever, never), and energy (kcal/d, continuous); AI, Adequate Intake; BMI, body mass index; CTT-1, Color Trails Test part 1; CTT-2, Color Trails Test part 2; CVLT-l, California Verbal Learning Test – long recall; CVLT-s, California Verbal Learning Test – short recall; CVLT-t, California Verbal Learning Test – sum; DST, Digit Span Test; ECW, extracellular water; GCF, Global Cognitive Function; GPT-d, Grooved Pegboard Test dominant hand; GPT-n, Grooved Pegboard Test non-dominant hand; Posm, plasma osmolality; TBW, total body water; UC, urine colour; Uosm, urine osmolality; USG, urine specific gravity; VFT, Verbal Fluency Tests; VT, Vocabulary Test

**Table 4:** Pearson correlation coefficients between hydration status and tests of cognitive function tests in men (r, *p*)

Variables	Water intake		Uosm		USG		UC		%TBW		ECW/TBW		Posm	
	Crude	Partial^a^	Crude	Partial^a^	Crude	Partial^a^	Crude	Partial^a^	Crude	Partial^a^	Crude	Partial^a^	Crude	Partial^a^
CVLT-t	-0.15 *p* = 0.638	-0.02 *p* = 0.968	0.08 *p* = 0.814	0.34 *p* = 0.503	0.30 *p* = 0.342	0.58 *p* = 0.223	0.57 *p* = 0.054	0.85 *p* = 0.034*	0.54 *p* = 0.068	0.37 *p* = 0.465	0.05 *p* = 0.874	0.27 *p* = 0.611	-0.02 *p* = 0.959	-0.26 *p* = 0.619
CVLT-s	-0.09 *p* = 0.784	0.30 *p* = 0.562	0.02 *p* = 0.941	0.27 *p* = 0.610	0.03 *p* = 0.929	0.01 *p* = 0.982	0.23 *p* = 0.469	0.43 *p* = 0.390	0.43 *p* = 0.161	-0.12 *p* = 0.826	-0.15 *p* = 0.639	-0.29 *p* = 0.579	0.12 *p* = 0.703	-0.23 *p* = 0.661
CVLT-l	0.01 *p* = 0.984	0.26 *p* = 0.619	-0.13 *p* = 0.681	0.47 *p* = 0.352	-0.03 *p* = 0.922	0.14 *p* = 0.794	0.18 *p* = 0.578	0.41 *p* = 0.425	0.51 *p* = 0.094	-0.11 *p* = 0.831	0.03 *p* = 0.936	-0.01 *p* = 0.992	0.16 *p* = 0.625	0.20 *p* = 0.699
CTT1	0.26 *p* = 0.417	0.73 *p* = 0.097	-0.13 *p* = 0.681	0.08 *p* = 0.880	-0.40 *p* = 0.202	-0.46 *p* = 0.364	-0.25 *p* = 0.431	-0.22 *p* = 0.679	-0.35 *p* = 0.261	-0.01 *p* = 0.978	0.27 *p* = 0.390	-0.36 *p* = 0.487	0.21 *p* = 0.505	-0.17 *p* = 0.744
CTT2	0.35 *p* = 0.272	0.57 *p* = 0.233	-0.21 *p* = 0.504	0.15 *p* = 0.771	-0.26 *p* = 0.406	-0.49 *p* = 0.320	-0.41 *p* = 0.180	-0.70 *p* = 0.124	-0.17 *p* = 0.605	-0.31 *p* = 0.554	0.34 *p* = 0.286	-0.19 *p* = 0.713	0.02 *p* = 0.947	0.40 *p* = 0.437
GPT-d	0.00 *p* = 0.996	0.49 *p* = 0.324	0.42 *p* = 0.173	0.44 *p* = 0.388	0.47 *p* = 0.127	0.58 *p* = 0.230	0.37 *p* = 0.232	0.61 *p* = 0.199	0.25 *p* = 0.440	0.95 *p* = 0.004*	0.55 *p* = 0.061	0.63 *p* = 0.177	-0.26 *p* = 0.423	-0.25 *p* = 0.636
GPT-n	-0.03 *p* = 0.914	0.35 *p* = 0.491	0.35 *p* = 0.265	0.42 *p* = 0.405	0.49 *p* = 0.106	0.72 *p* = 0.109	0.39 *p* = 0.209	0.63 *p* = 0.177	0.21 *p* = 0.517	0.95 *p* = 0.003*	0.62 *p* = 0.033*	0.68 *p* = 0.140	-0.24 *p* = 0.456	-0.33 *p* = 0.523
DST	-0.15 *p* = 0.642	-0.24 *p* = 0.646	-0.45 *p* = 0.145	-0.45 *p* = 0.365	-0.23 *p* = 0.464	-0.58 *p* = 0.225	-0.66 *p* = 0.019*	-0.93 *p* = 0.007*	-0.24 *p* = 0.448	-0.72 *p* = 0.107	-0.16 *p* = 0.615	-0.53 *p* = 0.284	0.36 *p* = 0.245	0.10 *p* = 0.846
VT	-0.44 *p* = 0.151	-0.58 *p* = 0.227	-0.11 *p* = 0.737	-0.13 *p* = 0.806	0.04 *p* = 0.909	0.14 *p* = 0.784	0.11 *p* = 0.725	0.29 *p* = 0.570	0.48 *p* = 0.111	-0.15 *p* = 0.780	-0.22 *p* = 0.491	0.07 *p* = 0.894	0.23 *p* = 0.478	0.18 *p* = 0.733
VFT	-0.14 *p* = 0.662	-0.14 *p* = 0.793	-0.41 *p* = 0.187	-0.12 *p* = 0.817	-0.23 *p* = 0.469	0.10 *p* = 0.845	0.25 *p* = 0.428	0.66 *p* = 0.151	0.41 *p* = 0.187	0.08 *p* = 0.879	-0.20 *p* = 0.527	-0.28 *p* = 0.593	0.31 *p* = 0.320	-0.59 *p* = 0.223
GCF	-0.28 *p* = 0.372	-0.65 *p* = 0.162	-0.32 *p* = 0.316	-0.32 *p* = 0.524	-0.15 *p* = 0.639	-0.12 *p* = 0.817	-0.03 *p* = 0.920	0.01 *p* = 0.985	0.21 *p* = 0.515	-0.54 *p* = 0.272	-0.53 *p* = 0.074	-0.34 *p* = 0.509	-0.08 *p* = 0.817	-0.61 *p* = 0.198

*p-value ≤ 0.05 ^a^In contrast to the crude (not adjusted) the partial correlation coefficients were adjusted for age (years, continuous), BMI (kg/m2, continuous), education (< 16 years, ≥ 16 years), smoking (ever, never), alcohol consumption (ever, never), and energy (kcal/d, continuous); AI, Adequate Intake; BMI, body mass index; CTT-1, Color Trails Test part 1; CTT-2, Color Trails Test part 2; CVLT-l, California Verbal Learning Test – long recall; CVLT-s, California Verbal Learning Test – short recall; CVLT-t, California Verbal Learning Test – sum; DST, Digit Span Test; ECW, extracellular water; GCF, Global Cognitive Function; GPT-d, Grooved Pegboard Test dominant hand; GPT-n, Grooved Pegboard Test non-dominant hand; Posm, plasma osmolality; TBW, total body water; UC, urine colour; Uosm, urine osmolality; USG, urine specific gravity; VFT, Verbal Fluency Tests; VT, Vocabulary Test

**Fig. 1:**
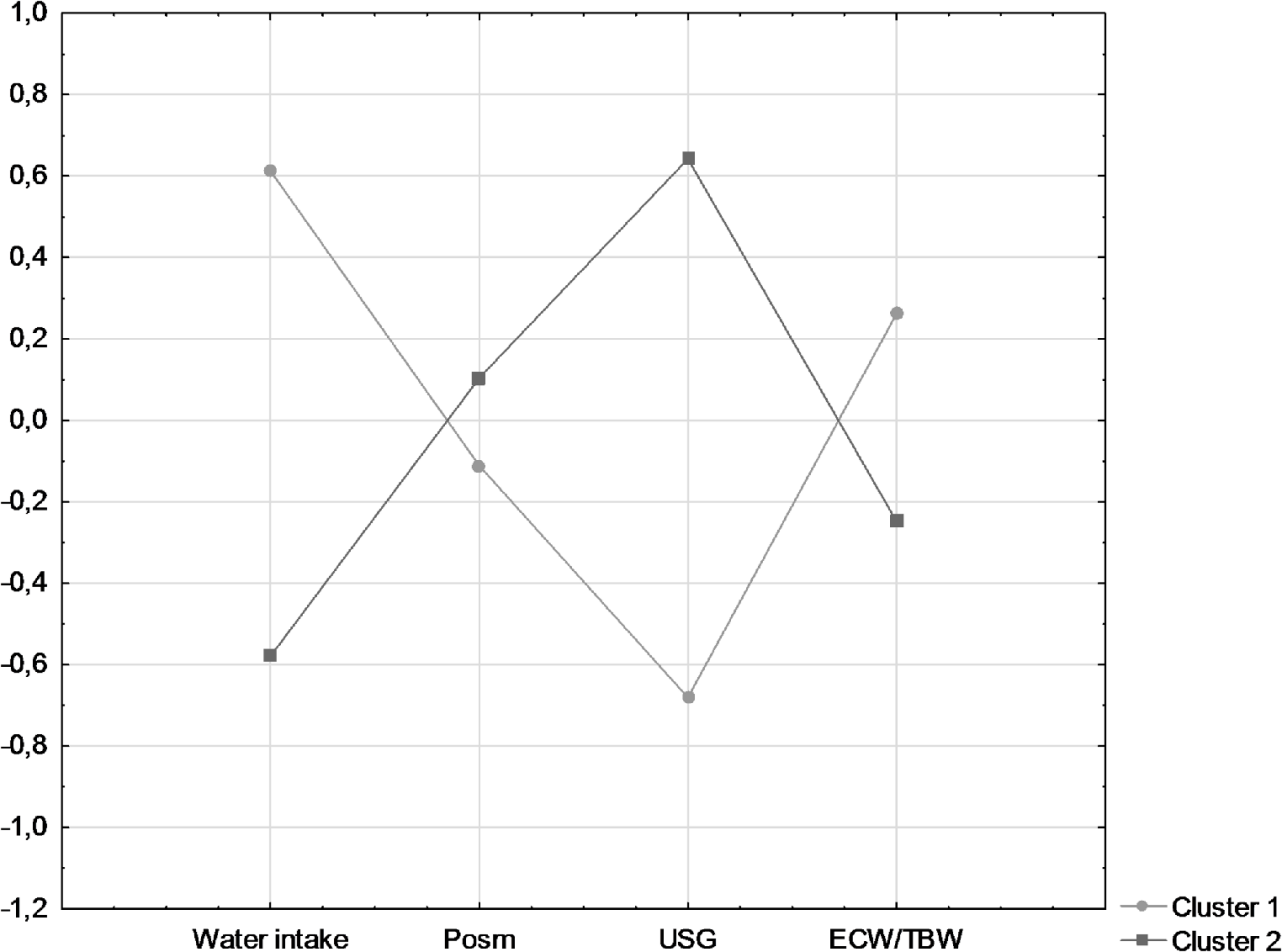
Hydration parameters characteristics by clusters analysis according to the input variables (Z-scores). Cluster 1: Characterized by higher water intake, lover plasma osmolality (Posm), lower urine specific gravity (USG), and higher Extracellular Water/Total Body Water ratio (ECW/TBW). Cluster 2: Characterized by lower water intake, higher plasma osmolality, higher urine specific gravity, and lower ECW/TBW ratio. All input variables were standardized to achieve a mean equal to 0 and a standard deviation equal to 1 and expressed as Z-score values. Significant differences were noted for water intake and USG. Cluster 1 represents a group with proper hydration, while Cluster 2 shows a different pattern, possibly indicating a potential low-intake dehydration

Cluster 1 included 17 participants (48.6%) and was characterized by higher water intake and AI% and lower Uosm, USG, UC, TBC, and ECW. In contrast, cluster 2, characterized by lower water intake and AI% and higher Uosm, USG, UC, TBC, and ECW, included 18 participants (51.4%) (Fig. 1). Cluster 2 was characterized by significantly higher results in language function tests, including VT (*p* = 0.041) and VFT (*p* = 0.041) (Table 5). There were no significant differences between the clusters in dietary nutrient intake (Table 6) or blood parameters, except for total cholesterol (*p* = 0.042), which was lower in cluster 2 (data not shown in tables).

**Table 5:** Results of hydration and cognitive function tests by cluster

Variables	Cluster 1 (*n* = 17)	Cluster 2 (*n* = 18)	*P*-value
Water intake *(g/d)*	3 063 ± 447.8	2 433 ± 407.6	< 0.001*
%AI	149.4 ± 23.9	107.8 ± 19.5	< 0.001*
Uosm *(mOsm/kg)*	347.7 ± 114.1	599.6 ± 174.0	< 0.001*
USG	1.01 ± 0.002	1.02 ± 0.01	< 0.001*
UC	3.82 ± 1.51	5.28 ± 1.35	0.008*
%TBW	47.1 ± 6.0	49.2 ± 6.9	0.382
TBC *(L)*	33.1 ± 6.0	37.9 ± 8.1	0.044*
ICW *(L)*	20.1 ± 3.6	23.1 ± 5.0	0.052
ECW *(L)*	13.0 ± 2.4	14.7 ± 3.1	0.036*
ECW/TBW	0.39 ± 0.01	0.39 ± 0.01	0.156
Posm *(mOsm/kg)*	292.6 ± 4.4	293.8 ± 6.0	0.717
CVLT-t *(pt.)*	57.5 ± 7.8	57.1 ± 9.7	0.961
CVLT-s *(pt.)*	11.2 ± 2.3	11.9 ± 2.9	0.468
CVLT-l *(pt.)*	12.0 ± 1.9	12.4 ± 2.2	0.531
CTT1 *(sec.)*	57.8 ± 13.6	55.5 ± 15.3	0.779
CTT2 *(sec.)*	115.9 ± 39.8	107.4 ± 28.1	0.704
GPT-d *(sec.)*	77.1 ± 10.8	76.9 ± 21.9	0.255
GPT-n *(sec.)*	87.1 ± 11.4	85.3 ± 21.7	0.478
DST *(pt.)*	10.3 ± 2.5	11.7 ± 3.3	0.248
VT *(pt.)*	29.8 ± 14.2	39.8 ± 10.5	0.041*
VFT *(pt.)*	80.1 ± 14.7	92.6 ± 19.3	0.041*
GCF	-0.153 ± 0.545	0.144 ± 0.572	0.053

*p-value ≤ 0.05 (Mann–Whitney *U* test); AI, Adequate Intake; BMI, body mass index; CTT-1, Color Trails Test part 1; CTT-2, Color Trails Test part 2; CVLT-l, California Verbal Learning Test – long recall; CVLT-s, California Verbal Learning Test – short recall; CVLT-t, California Verbal Learning Test – sum; DST, Digit Span Test; ECW, extracellular water; GCF, Global Cognitive Function; GPT-d, Grooved Pegboard Test dominant hand; GPT-n, Grooved Pegboard Test non-dominant hand; Posm, plasma osmolality; pt., points; sec., seconds; TBW, total body water; UC, urine colour; Uosm, urine osmolality; USG, urine specific gravity; VFT, Verbal Fluency Tests; VT, Vocabulary Test

**Table 6:** Characteristics of the study group by cluster (mean ± SD)

Variables	Cluster 1 (*n* = 17)	Cluster 2 (*n* = 18)	*P*-value
Sex *(%)*			
* Women*	62.2	34.8	0.006 ^b*^
* Men*	16.7	83.3	
Age *(years)*	69.0 ± 4.8	68.5 ± 3.5	0.741 ^a^
BMI *(kg/m*^*2*^*)*	27.3 ± 5.0	27.4 ± 3.3	0.729 ^a^
Physical activity *(%)*			
* Low*	44.4	55.6	0.878 ^b^
* Moderate*	53.3	46.7	
* High*	50	50	
Regular smoking *(%)*	40	60	0.380 ^b^
Alcohol consumption *(%)*	46.7	53.3	0.858 ^b^
Supplement use *(%)*	51.7	48.3	0.412 ^b^
Drugs use *(%)*	42.3	57.7	0.208 ^b^
Chronic diseases (%)	46.7	53.3	0.581 ^b^
Education time *(%)*			
* < 16 years*	66.7	33.3	0.122 ^b^
* ≥ 16 years*	39.1	60.9	
Residence *(%)*			
* Urban area*	50	50	0.324 ^b^
* Suburban area*	0	100	
Lives alone *(%)*	64.3	35.7	0.233 ^b^
Social contacts *(%)*	48.6	51.4	0.485 ^b^
Energy intake *(kcal/d)*	1644 ± 364	1778 ± 492	0.499 ^a^
Sodium intake *(mg/d)*	3319 ± 980	3367 ± 1290	0.961 ^a^
Potassium intake *(mg/d)*	3279 782	3089 876	0.347 ^a^
Magnesium intake *(mg/d)*	359 100	361 127	0.934 ^a^
Calcium intake *(mg/d)*	619 207	701 236	0.330 ^a^
Protein intake *(g/d)*	73.5 ± 23.2	80.9 ± 23.9	0.499 ^a^
Fat intake *(g/d)*	67.0 ± 19.1	69.0 ± 20.7	0.934 ^a^
Carbohydrate *(g/d)*	194 ± 48.3	216 ± 84.2	0.632 ^a^

*p-value ≤ 0.05 (^a^ - Mann–Whitney *U* test, ^b^ - Chi^2^ test)

BMI – body mass index

## Discussion

Although this was a pilot study with a small group of subjects, it provided interesting observations about the relationship between hydration status and cognitive functions. Research in this area is inconsistent. A meta-analysis of studies in healthy young adults shows that a loss of more than 2% of body weight impairs attention, executive functions, and motor coordination, but not information processing, memory, or reaction time [12]. Other meta-analyses did not confirm these results, suggesting dehydration, compared to proper hydration, may not significantly impair cognitive performance or any cognitive domain [11]. Inadequate water intake and dehydration have been documented as a cause or an effect of dementia in older adults in long-term care facilities [27, 28]. It remains unclear whether and how the body’s hydration status under typical everyday conditions is associated with changes in cognitive functions. In previous research, total water intake and adherence to the European Food Safety Authority (EFSA) recommendations were not associated with cognitive function [29, 30], which is also confirmed by our results.

Until now, studies have only used a single method to assess hydration status. Two studies published so far have assessed hydration status using urine parameters. A prospective study was conducted with 17 active men experienced in mountain hiking. Group 1 (younger; mean age 24 ± 3 years, *n* = 9) and group 2 (older; 56 ± 4 years, *n* = 8) for 10 days intensively marched in the mountains in April in the Scottish Highlands. A significant association was found between increased urine osmolality and a slower reaction time. Reaction time was increased in both groups, but the older group showed a marked increase in dehydration. In addition, the older group had lower perceptions of thirst than the younger group. The authors suggested that impaired ability to regulate body temperature may be more remarkable in older participants [31]. Another study among 60 free-living volunteers aged 60–93 showed no statistically significant relationships between urine specific gravity and cognitive functions [32]. In this study, urine osmolality, specific gravity, and color did not correlate with the results of cognitive test scores in the total group; however, there was a positive correlation between urine parameters and the memory/learning test in women and men.

Two recent studies have assessed hydration status by determining total body water content using bioelectrical impedance. In a study of 28 participants (22 women) aged 50 to 82, a lower %TBW/kg was significantly associated with slower psychomotor processing speed and impaired memory/attention [33]. Another study with 21 postmenopausal women (aged 50 to 78) examined the relationship between total body water, extracellular and intracellular water, vs. declarative memory and working memory. Again, higher levels of TBW were associated with better memory/learning and working memory [34]. In this study, the higher percentage of total body water was statistically significantly associated with poorer memory/learning test (CVLT), verbal fluency test (VFT), and overall cognitive function (GCF) results in women and processing speed test in men, which is contrary to previous research results.

In two studies, hydration status was assessed by calculated serum osmolarity (Sosm). In this study, Posm did not correlate with the results of the cognitive test scores. In a study of 2,506 subjects aged ≥ 60, a curvilinear relationship was observed between attention and processing speed and Sosm in women. Both high and low levels of Sosm were associated with lower test scores. The authors suggested that those in the lowest Sosm category may have been overhydrated, potentially due to the age-related decline in the kidney’s ability to excrete water, leading to hyponatremia, which may be independently associated with an increased risk of cognitive impairment. In contrast, higher levels of Sosm in men were associated with significantly better results in verbal learning and memory tests. This was explained by differences in the thresholds at which men and women begin to feel the effects of insufficient hydration. Additionally, the point at which an individual experiences discomfort from thirst can impact their attention, concentration, and capability to execute complex tasks [30]. A prospective cohort study of 6,874 adults with metabolic syndrome showed that poorer hydration status (measured by plasma osmolality) was associated with greater global cognitive decline over a 2-year period, especially in men. When each neuropsychological test was analyzed separately, there was an increase in the digit repetition test [29].

Despite the disparities in results, both studies with large subject groups highlight the differences between women and men. Furthermore, the studies suggest a protective effect of adequate hydration against cognitive impairment/deterioration, although this association was not found in all cognitive domains investigated.

The limitation of this study was the small number of subjects. To date, there have been no studies using multiple parameters of hydration status, so we decided to prepare this pilot study. Another limitation of the study was the reliance on self-reported food and fluid intake data. However, this 3-day food record method offers significant advantages over alternative approaches. In contrast to food frequency questionnaires, the food record method minimizes reliance on memory, as participants record their intake immediately after consumption [35]. The literature suggests that food records should be long enough to provide reliable information about usual intake but balanced against the risk of poor adherence if a record is too long. Recording periods longer than 4 consecutive days were considered unsatisfactory [36]. Data collected using the 3- or 4-day food record method is also recommended for assessing water intake [37]. Another limitation was the recruitment of subjects without symptoms of dehydration due to the willingness to include noninstitutionalized subjects. There is a lack of a dehydrated comparison group, which may explain the low incidence of dehydration in free-living older adults [34]. To our knowledge, this is the first study to compare several markers of hydration status and cognitive function tests. It should be emphasized that the markers used to assess hydration status were selected following the literature [9, 38–40]. Plasma osmolality was determined directly by a certified laboratory, urinalysis included several parameters, and the recommended resistance level was used to assess body water content during body composition measurements. Another strength of the study is the participation of a very homogeneous group of older people in terms of age, education, physical and social activity, cognitive function (without cognitive disorders), and hydration.

## Conclusions

The results obtained are not fully consistent with other studies; however, significant relationships between some indicators of hydration status and selected cognitive domains were observed. This pilot study complements previous research on the relationship between hydration status and cognitive function in older adults, emphasizing that even small changes in hydration status assessment parameters can affect cognitive outcomes. This pilot study indicates the need for further research on hydration and cognitive function. It should be determined whether plasma osmolality is a more recommended measure of hydration status than urinary biomarkers among older adults, taking into account the assessment of cognitive functioning and risk of cognitive decline. The importance of the balance between the amount of water consumed and excreted should also be assessed, as the relationship between body water retention and cognitive function is unclear. The long-term effect of low water intake should be evaluated in a larger study group.

## Data Availability

All data will be available upon a reasonable request to the corresponding author.
